# Commonalities of platelet dysfunction in heart failure with preserved ejection fraction and underlying comorbidities

**DOI:** 10.1002/ehf2.15090

**Published:** 2024-10-07

**Authors:** Giorgia D'Italia, Blanche Schroen, Judith M.E.M. Cosemans

**Affiliations:** ^1^ Department of Biochemistry, Cardiovascular Research Institute Maastricht (CARIM) Maastricht University Maastricht The Netherlands; ^2^ Department of Physiology, Cardiovascular Research Institute Maastricht (CARIM) Maastricht University Maastricht The Netherlands

**Keywords:** platelets, heart failure with preserved ejection fraction, atrial fibrillation, diabetes mellitus, obesity, ageing, hypertension, sex

## Abstract

Heart failure with preserved ejection fraction (HFpEF) is characterized by a lack of a specific targeted treatment and a complex, partially unexplored pathophysiology. Common comorbidities associated with HFpEF are hypertension, atrial fibrillation, obesity and diabetes. These comorbidities, combined with advanced age, play a crucial role in the initiation and development of the disease through the promotion of systemic inflammation and consequent changes in cardiac phenotype. In this context, we suggest platelets as important players due to their emerging role in vascular inflammation. This review provides an overview of the role of platelets in HFpEF and its associated comorbidities, including hypertension, atrial fibrillation, obesity and diabetes mellitus, as well as the impact of age and sex on platelet function. These major HFpEF‐associated comorbidities present alterations in platelet behaviour and in features linked to platelet size, content and reactivity. The resulting dysfunctional platelets can contribute to further increase inflammation, oxidative stress and endothelial dysfunction, suggesting an active role of these cells in the initiation and progression of HFpEF. Recent evidence shows that reduced platelet count and elevated mean platelet volume are associated with worsening heart failure in HFpEF patients. However, the specific mechanisms by which platelets contribute to HFpEF development and progression are still largely unexplored, with only a few studies investigating platelet function in HFpEF. We discuss the limited yet significant body of research investigating platelet function in HFpEF, emphasizing the need for more comprehensive studies. Additionally, we explore the potential mechanisms through which platelets may influence HFpEF, such as their interactions with the vascular endothelium and the secretion of bioactive molecules like cytokines, chemokines and RNA molecules. These interactions and secretions may play a role in modulating vascular inflammation and contributing to the pathophysiological landscape of HFpEF. The review underscores the necessity for future research to elucidate the precise contributions of platelets to HFpEF, aiming to potentially identify novel therapeutic targets and improve patient outcomes. The evidence presented herein supports the hypothesis that platelets are not merely passive bystanders but active participants in the pathophysiology of HFpEF and its comorbidities.

## Introduction

Heart failure (HF) with preserved ejection fraction (HFpEF) is the most common form of HF and is considered a systemic disease. Commonly associated comorbidities include systemic hypertension, obesity, diabetes and atrial fibrillation (AF). Unlike HF with reduced ejection fraction (HFrEF), HFpEF lacks a specific targeted treatment, focusing instead on improving quality of life and preventing hospitalization by managing associated comorbidities.^1^

In recent years, landmark clinical trials have evaluated the efficacy of sodium–glucose cotransporter‐2 (SGLT2, *SLC5A2*) inhibitors, a class of antidiabetic medication, in treating HFpEF. The EMPEROR‐Preserved trial showed that the SGLT2 inhibitor empagliflozin reduced the risk of HF hospitalization or cardiovascular death in HFpEF patients.^2^ Similarly, the DELIVER trial demonstrated that the SGLT2 inhibitor dapagliflozin reduced the risk of worsening HF or cardiovascular death in HFpEF and HF with mid‐range ejection fraction (HFmrEF) patients.^3^ These results led to the incorporation of SGLT2 inhibitors into international guidelines for the HFpEF treatment.^4^

Comorbidity prevalence varies among HFpEF cohorts. Hypertension is prevalent in ~75% of HFpEF patients.^5^ There is also a known strong association between HFpEF and AF: On one side, AF is a crucial risk factor for the development of HFpEF, while on the other side, HFpEF patients have a higher risk of developing AF. Approximately half of the patients with HFpEF also present AF.^5^ Type 2 diabetes mellitus (DM), present in around 30% of HFpEF patients, increases the risk of hospitalization and mortality and can contribute to left ventricular dysfunction development.^6^ Furthermore, HFpEF patients are typically elderly, mostly female and obese.^5^

Besides the role of comorbidities in the prognosis and treatment of HFpEF, they also play a crucial role in disease development, but the specific mechanisms involved are still under active investigation. The paradigm for HFpEF proposes that the different comorbidities can induce a systemic pro‐inflammatory state, which can promote microvascular dysfunction (MVD) and affect the coronary endothelial cells, increasing oxidative stress and decreasing endothelium‐derived nitric oxide (NO) bioavailability. The limited NO generation can directly affect the adjacent cardiomyocytes, contributing to the development of cardiomyocyte hypertrophy and left ventricular structural remodelling and increased stiffness.^7^ In this sequence of events, MVD plays a key role.^8^ In the past years, platelets emerged as players of significance in vascular integrity and inflammation, thanks to their ability to interact with—among others—endothelial cells and through their release of inflammatory mediators like cytokines and chemokines, as well as the release of (small) RNA molecules.^9^, ^10^, ^11^ While few studies investigate platelet functions in HFpEF, their role in associated comorbidities is well‐studied. This review provides a comprehensive overview of platelet behaviour in common HFpEF‐linked comorbidities, focusing on activation, morphology, RNA content and microvesicle release. These platelet characteristics and their biological relevance can be investigated through different basic research laboratory techniques, as summarized in Table 1, which can help as a guide throughout the text.

**Table 1:** Guide to the study of platelet phenotype and function through basic research laboratory techniques.

Platelet characteristics	Parameters	Most commonly used laboratory techniques	Biological relevance
Platelet production	Reticulated platelet population (%) Immature platelet fraction (IPF) (%) Thrombopoietin (TPO) (concentration)	Flow cytometry Automated blood analyser ELISA	Reticulated platelets are ‘young’ and immature platelets: larger, RNA‐richer. Their elevated levels indicate increased platelet turnover.^12^ TPO regulates platelet production and is important in the diagnosis and management of haematological disorders.^13^
Platelet morphology	Mean platelet volume (MPV) (fL) Platelet distribution width (PDW) (%) Intracellular structures, cytoskeleton Lipid profile	Automated blood analyser Automated blood analyser Microscopy Mass spectrometry	The MPV and PDW indices measure platelet volume. Increased platelet volume indicates higher platelet reactivity, potentially increasing the risk of adverse cardiovascular events.^14^ Changes in intracellular structures and membrane composition can affect platelet activation, surface receptor expression and microvesicle release.^15^
Platelet function	Aggregation in response to agonists (%) Surface receptor expression (glycoproteins, integrins, procoagulant platelets) (MFI, %) Granule secretion (CD62P, CD63, ATP) (MFI, %) Thrombus formation in real‐time, platelet adhesion in response to agonists, the effect of shear (multiple parameters) Platelet spreading on agonist‐coated surface Mitochondrial function	Light transmission aggregometry (LTA) Flow cytometry Flow cytometry/Lumi aggregometry Microscopy/microfluidics Microscopy Flow cytometry/respirometers	Ex vivo platelet activation/reactivity measurements explore various aspects of platelet function in response to agonist stimulation. Increased reactivity involves enhanced platelet aggregation, surface receptor expression, degranulation, adhesion and formation of large, active thrombi. Abnormalities in platelet reactivity may indicate bleeding disorders or heightened thrombosis risk.^16^
Platelet intracellular signalling	Intraplatelet calcium kinetics Protein expression/phosphorylation Transcriptome profile Proteomic profile	Flow cytometry/microplate reader Western blot RNA sequencing, RT‐qPCR Mass spectrometry	Assessing platelet calcium kinetics, protein expression and phosphorylation offers insights into the molecular mechanisms of platelet activation.^17^
Platelet in vivo activation	Circulating platelet activation markers (β‐TG, PF4, CD62P, CD40L, TXB_2_, PDGF, CD63, serotonin) (concentration) Platelet‐derived microvesicle (PMV) count and characterization (concentration, size) Urinary/serum TXA_2_ metabolites (concentration)	ELISA Flow cytometry/microscopy/nanoparticle tracking analyser Radioimmunoassay/ELISA	Activated platelets release granule contents and microvesicles containing various molecules, including miRNAs.^18^ Elevated circulating platelet markers indicate increased platelet activation. Measuring TXA_2_ metabolites provides information about processes involving the arachidonic acid pathway and platelet activation.^19^

Abbreviations: ELISA, enzyme‐linked immunosorbent assay; MFI, mean fluorescence intensity; miRNAs, microRNAs; RT‐qPCR, quantitative reverse transcription polymerase chain reaction; TXA_2_, thromboxane A_2_; TXB_2_, thromboxane B_2_; β‐TG, β‐thromboglobulin.

## Methods

A literature search was conducted on PubMed over the period January 1970–May 2024 using the following keywords: Heart Failure with Preserved Ejection Fraction, Platelets, Diabetes mellitus, Obesity, Hypertension, Atrial fibrillation, Sex and Ageing. Only publications containing quantifiable data on platelet characteristics and/or function were included. Artificial intelligence (AI) technology was used for spell and grammar check.

## Platelet pathophysiology in HFpEF: Insufficiently explored

Until now, relatively few studies investigated platelet phenotype and function specifically related to HFpEF. The MyoVasc study (*n* = 637 HFpEF and *n* = 342 HFrEF) was the first to explore platelet indices, cardiac function and outcomes in both HFpEF and HFrEF patients. Increased mean platelet volume (MPV) and lower count were associated with the worsening of HF as a composite of progression from asymptomatic to symptomatic HF, hospitalization due to HF worsening, and cardiac death in asymptomatic and symptomatic HF subjects. Notably, MPV > 75th percentile and platelet count <25th percentile had a stronger impact on HF worsening in HFpEF than in HFrEF, independent of age and sex and after correction for common cardiovascular risk factors and cancer. This suggests a potentially significant role for platelets in HFpEF pathophysiology.^20^ Recently, another study found increased MPV values in HFpEF patients compared with age‐ and sex‐matched controls, and high MPV to be an independent predictor of poor outcome, higher risk of all‐cause mortality or first HF hospitalization, in these patients.^21^

As a follow‐up on the initial MyoVasc study,^20^ the same group studied sex‐specific associations between parathyroid hormone (PTH)—which is associated with all‐cause and cardiovascular mortality in HF patients^22^—and platelet indices in different HF phenotypes.^23^ The authors found an inverse association between PTH and platelet count in both males and females with HFpEF and males with HFrEF. PTH and MPV are positively associated between PTH and MPV in females with HFrEF, suggesting a sex‐specific mechanism in the interaction between PTH and platelets.^23^ HFpEF and HFrEF can be considered as distinct syndromes with different associated comorbidities, treatment and aetiology, as reviewed by Simmonds *et al*.^24^ HFrEF is a large vessel disease, often resulting from chronic cardiac remodelling after a myocardial infarction (MI) or because of a hereditary cardiomyopathy. HFpEF, on the other hand, is driven by microvascular disease. We have previously reviewed the role of platelets in large vessels, focusing on their role in atherogenesis and in MI.^11^

Platelet activation has been described in patients with congestive HF versus healthy controls as increased whole blood aggregation and increased markers of platelet activation markers, such as soluble P‐selectin (CD62P, *SELP*), β‐thromboglobulin [cleavage product from platelet basic protein (*PPBP*)] and CD40L (*CD40LG*), and a higher MPV, as reviewed by Mentz *et al*.^25^ Interestingly, patients with congestive HF tend to have similar levels of platelet markers compared with patients with coronary disease without HF.^25^ Conflicting data have been observed with regard to the association of platelet indices, activation markers and functional measurements with prognosis.^25^, ^26^

To our knowledge, until now, only two small cohort studies investigated platelet function in HFpEF, focusing on platelet NO responses and mitochondrial function. NO, released by the endothelium, is an important inhibitor of platelet activation, stimulating soluble guanylyl/guanyl cyclase (sGC) and subsequent production of the second messenger, cyclic guanosine 3′,5′‐monophosphate (cGMP). cGMP then activates protein kinase G (PKG) and downstream pathways leading to platelet inhibition.^27^ In patients with both HFpEF and chronic AF, platelets displayed impaired responses to NO due to sGC dysfunction.^28^ These results suggest the presence of platelet resistance to inhibitory action mediated by NO, which can imply platelet abnormal activation in HFpEF patients. In another study on patients with HFpEF and pulmonary hypertension, an alteration in platelet bioenergetic activity was observed, with a change in platelet mitochondrial function and increased respiratory capacity.^29^

The current understanding of platelet behaviour in HFpEF suggests altered characteristics, impaired response to antiplatelet agents and dysfunctional bioenergetic activation, thus suggesting aberrant platelet activation in these patients. We hypothesize that these alterations may directly impact platelet interactions with the vessel wall, potentially promoting inflammation and endothelial dysfunction, thus contributing to HFpEF initiation and progression. Current knowledge assesses platelet function through indirect markers of platelet activation, while the actual measurement of platelet activation by functional assays is currently lacking. In addition, the study of the interaction between platelets and endothelium in the HFpEF context still needs to be investigated. This could provide answers on the possible role of platelets in MVD in HFpEF and consequences of aberrant platelet activation on patient outcome.

In contrast to the current limited knowledge about platelets in HFpEF specifically, multiple platelet abnormalities are observed in HFpEF comorbidities, including hypertension, AF, obesity and DM. Additionally, age and sex impact on platelet functions, coinciding with HFpEF, which predominantly affects the elderly population and exhibits a higher prevalence among females.

The following paragraphs describe the current knowledge of platelet‐altered phenotypes and behaviour in the most commonly encountered HFpEF comorbidities, as well as the impact of age and sex.

## Platelets in hypertension

Hypertension is the most prevalent comorbidity in HFpEF patients.^5^ It is a major cause of premature death and is associated with an increased incidence of AF, heart and renal failure, and stroke.^30^ Persistent high blood pressure contributes to vascular remodelling, inflammation and endothelial dysfunction,^31^ fostering platelet dysfunction and thrombotic risk.

### Platelets in hypertension present morphological and functional alterations

Multiple studies suggest increased platelet activation to be associated with high blood pressure, correlating with thrombotic risk.^32^ Therefore, investigating various aspects of platelet function and behaviour in hypertension is crucial. Platelet abnormalities can be assessed through multiple methods (see Table 1), including measuring changes in morphological characteristics such as volume and mass. In hypertension, platelets show increased volume indices, including MPV and platelet distribution width (PDW).^33^, ^34^, ^35^ Functional assays assessing platelet reactivity to various agonists can also provide insight, with hypertensive patients often showing enhanced platelet adhesion and aggregation.^36^, ^37^, ^38^, ^39^ Additionally, measuring platelet markers released by granules into circulation reflects platelet in vivo activation, with hypertensive individuals displaying higher plasma β‐thromboglobulin levels compared with healthy individuals.^36^

Alterations in platelet function may result from changes in intracellular signalling. Activation of platelet receptors by various stimuli triggers complex signalling pathways, leading to increased intracellular calcium, which drives activation responses such as platelet shape changes and granule release.^17^ In hypertensive patients, increased levels of platelet intracellular calcium have been observed, as reviewed by El Haouari and Rosado.^40^ These changes may be driven by oxidative stress, where increased reactive oxygen species (ROS) stimulate protein tyrosine phosphorylation, inducing calcium mobilization and thromboxane A_2_ (TXA_2_) synthesis.^40^ Furthermore, ROS can deactivate the inhibitory NO/cGMP pathway, promoting platelet activation. Platelets from hypertensive individuals often exhibit reduced NO levels, with males showing greater reduction compared with females.^41^ Moreover, platelets from hypertensive individuals show reduced activation of NO synthase upon agonist stimulation, suggesting a defect in NO production.^39^ These deficiencies in the NO pathway may lead to reduced inhibition of platelet activation in circulation, contributing to platelet hyperactivation in hypertensive patients.

The platelet external membrane undergoes significant changes upon activation, potentially affected by pathological conditions. Hypertensive platelets exhibit increased P‐selectin expression and alterations in membrane lipid composition, including elevated phosphatidylserine (PS) content and reduced cholesterol content, potentially leading to increased membrane fluidity.^42^ The increased PS content is of interest given the essential role of PS in promoting blood coagulation.^43^ Recent studies highlight Piezo‐type mechanosensitive ion channel component 1 (Piezo‐1, *PIEZO1*) involvement in platelet activation in hypertension. Piezo1, present on the cell surface and opened upon mechanical pressure (e.g., high‐shear stress) allowing the entrance of calcium ions, was found to promote platelet activation in hypertensive mice.^44^ These results point out Piezo‐1 as an interesting candidate for inhibiting platelet activation under high‐shear conditions.

### Platelet‐derived microRNAs (miRNAs) and microvesicles: Their emerging role in hypertension

Despite lacking a nucleus, platelets contain functional miRNAs—small single‐stranded RNA molecules about 21–23 nucleotides long. These miRNAs can be transferred to other cell types via platelet‐derived extracellular vesicles, known as platelet‐derived microvesicles (PMVs), and can influence recipient cell function.^45^ Interestingly, although platelet miRNA and total RNA levels are lower than those of nucleated cells, platelets have higher miRNA/total RNA ratios compared with nucleated circulating cells like T cells.^46^ In vitro studies have demonstrated PMV‐mediated transfer of specific miRNAs (miR‐22, miR‐185, miR‐423‐5, miR‐320b and miR‐223) from platelets to endothelial cells, impacting cell‐specific functions by down‐regulating target genes like endothelial cell intercellular adhesion molecule‐1 (ICAM‐1, *ICAM1*)^47^ and insulin‐like growth factor 1 receptor (*IGF1R*).^48^ PMVs and their derived miRNAs are emerging as important in platelet communication with the vessel wall and immune cells, contributing to endothelial inflammation and leucocyte recruitment.^9^, ^10^ Hypertension is associated with increased circulating PMV levels,^49^ and PMVs isolated from hypertensive rats promote endothelial cell proliferation via miR‐142‐3p action.^50^ Importantly, reduced platelet miR‐22 and miR‐223 levels in hypertensive patients negatively correlate with systolic blood pressure and emerge as strong prognostic markers for cardiovascular disease (CVD).^51^

Taken together, altered platelet phenotype has been implicated in hypertension, with PMVs and their miRNA content impacting vascular cellular functions and longer term cardiovascular outcomes.

## Platelets in AF

AF, characterized by chaotic and irregular heart rhythms, is linked with HFpEF, exacerbating both morbidity and mortality outcomes when occurring together.^5^ Risk factors for AF, including ageing, hypertension, obesity, HF, coronary artery disease (CAD) and unhealthy lifestyle,^52^ overlap significantly with those for HFpEF. Moreover, both conditions share progressive left atrial myopathy driven by these cardiovascular risk factors.^5^ AF is associated with an increased risk of stroke and thrombo‐embolism.^52^ Subsequent sections will explore platelet morphological and functional implications in AF.

### Platelet phenotype in AF: Conflictual results

#### Platelet morphological changes in AF

Platelet morphological tests offer promising opportunities in predicting the prognosis of AF patients. The meta‐analysis conducted by Weymann *et al*. reveals that AF patients on average exhibit a lower platelet count than individuals in sinus rhythm (45 studies), an elevated MPV (19 studies) and decreased PDW (3 studies).^53^ Significant heterogeneity among the studies was observed in platelet count, MPV and PDW.^53^ MPV is regarded as a potential new predictor of the risk of ischaemic stroke, in combination with the CHA2DS2‐VASc score.^54^ The latter is in line with the finding that MPV and PDW inversely correlate with the international normalized ratio (INR),^55^ which is a ratio based on the results of a patient's prothrombin time and is used to monitor patients who are being treated with vitamin K antagonists.

Thrombocytopenia—a too low blood platelet count—is common in AF patients^53^, ^56^ and significantly associated with mortality.^56^ Finally, the proportion of reticulated platelets, so‐called young platelets, larger and RNA‐richer, is increased in AF, suggesting that platelets are more prone to activation. Interestingly, reticulated platelet content decreased after catheter ablation and return to sinus rhythm.^57^

#### Platelet activation in AF

AF is characterized by a prothrombotic state. The left atrial appendage is known as the main site of intra‐atrial thrombus formation,^52^ and for this reason, it is important to evaluate local coagulation and platelet activation and function in AF in blood samples directly obtained from the atria. In line, a study^58^ investigated platelet activation in left atrial blood samples from paroxysmal (*n* = 21) and persistent (*n* = 16) AF patients. The results showed reduced platelet aggregation in response to  thrombin receptor activating peptide (TRAP) in all AF patients compared with controls in sinus rhythm, while there were no differences in αIIbβ3 and GPIb receptor expression and P‐selectin expression among the groups. The decreased platelet basal aggregation to TRAP may result from protease‐activated receptor‐1 (PAR‐1) desensitization due to hypercoagulability. Notably, an acute episode of induced AF was able to partially reverse the decreased platelet response to PAR‐1 activation, suggesting a dynamic balance between platelet activation and desensitization in AF.^58^ A study by Willoughby *et al*. observed increased platelet P‐selectin expression (10.2 vs. 8.6%) and ADP‐induced aggregation in blood sampled from the left atrium compared with the right atrium measured by whole blood flow cytometry.^59^ At present, it is unknown if there is a biological relevance of a difference of 1.6% in absolute numbers in P‐selectin expression. Measuring multiple markers and using multiple techniques, in combination with clinical parameters, are recommended. In the Willoughby *et al*. paper,^59^ integrin αIIbβ3 (glycoprotein IIB/IIIa, *ITGA2B*, *ITGB3*) activation in flow cytometry was normal between groups and no aggregation traces or percentage of aggregation were provided, which, combined with the low number of participants (19), makes it difficult to draw solid conclusions regarding platelet reactivity in patients studied. In line with this, another study found oppositely significantly higher P‐selectin levels in the right versus the left atrium (60.3 vs. 59.3 ng/mL),^60^ but again, the question can be raised how meaningful this significant difference is biologically. In addition, there is evidence indicating systemic alterations in platelet function in AF patients. However, these data derive from studies with low patient numbers (<20) and are conflicting.^61^, ^62^

Circulating biomarkers inform on in vivo platelet activation in the patient: Upon activation, platelets release granule contents, soluble factors and PMVs into the bloodstream. Increased plasma levels of platelet activation markers, including β‐thromboglobulin, P‐selectin, CD40 ligand and soluble platelet glycoprotein V (sGPV, *GP5*), are observed in patients with AF as compared with controls.^63^ In line, levels of PMVs are increased in plasma from AF patients.^64^, ^65^ Moreover, extracellular vesicles predominantly derived from platelets were found in blood from the left atrial appendices of patients with AF versus non‐AF.^66^ Evidence on circulating soluble P‐selectin levels is less congruent.^63^ In addition, a study found decreased expression levels of platelet‐derived MiR‐150 in plasma and platelets isolated from patients with AF and HFrEF, compared with those with exclusively HFrEF.^67^

Overall, the evidence for altered platelet activation in AF, whether local or systemic, remains inconclusive. Despite findings of increased circulating platelet activation markers in AF patients, further research is necessary to understand the potential consequences of altered platelet phenotype and its possible impact on AF initiation and progression.

## Platelets in obesity

Obesity, defined by a body mass index (BMI) ≥30 kg/m^2^, affects over 650 million people worldwide^68^ and is common in HFpEF patients. It is a key factor underlying the pathogenesis of several diseases, including diabetes. The majority of obese individuals also exhibit metabolic syndrome, defined by three or more risk factors: abdominal obesity, high blood glucose and triglycerides, low high‐density lipoprotein (HDL) and hypertension.^69^

### Altered platelet phenotype in obesity

Generally, obese individuals exhibit platelet hyperactivation. Obese female individuals have a higher platelet count compared with non‐obese female subjects.^70^ Morphologically, platelet volume (MPV) is increased in obese individuals, positively correlating with BMI.^71^ Larger platelets may contain more receptors and granules, implying increased platelet susceptibility to activation. Notably, in a group of >30 000 individuals, a high BMI was found to be positively associated with platelet count and the number of reticulated platelets,^72^ which is thought to imply younger and more reactive platelets. This suggests alterations in platelet production and/or clearance in obesity. The bone marrow microenvironment contains adipocytes, and high‐fat diet‐induced obesity has been shown to increase medullar adiposity in mice, linked to enhanced megakaryocyte maturation and proplatelet formation.^73^ This research topic and its impact for human (patho)physiology are gaining interest in the field.

Evidence indicates that obese insulin‐resistant individuals exhibit higher plasma levels of soluble P‐selectin and CD40L, both markers of platelet activation.^74^, ^75^ Furthermore, obesity is associated with increased expression of the platelet glycoprotein VI (GPVI, *GP6*) receptor, higher aggregation in response to GPVI stimulation and hyperactivation of downstream signalling.^76^ In addition, platelets isolated from obese subjects show a reduced sensitivity to endothelium‐derived NO and prostaglandin I_2_ (PGI_2_), suggesting an increased platelet activation tendency.^77^

Emerging tools such as platelet phosphoproteomics and lipidomic analysis reveal significant alterations in platelet biology in obesity, including activation of key signalling pathways like Src family kinase (SFK) and (hemi‐)immunoreceptor tyrosine‐based activation motif (ITAM) signalling, as well as pathways involved in microvesicle formation.^78^ Notably, the hyperactive state of platelets in obesity is characterized by a distinct lipid fingerprint, including decreased platelet cholesterol levels.^79^

It is becoming evident that platelets are not uniform, with distinct populations serving various roles in haemostasis and inflammation.^43^, ^80^ Obese non‐human primates have increased levels of a population of so‐called procoagulant platelets, caused by oxLDL‐enhanced GPVI signalling.^81^ This highlights the up‐regulation of specific platelet populations in disease, suggesting a possible targeted medical treatment of these specific platelet subtypes.

### Dysfunctional adipose tissue and leptin as a driver of prothrombotic changes, increased inflammation and oxidative stress

Dysfunctional adipose tissue triggers systemic inflammation by releasing inflammatory cytokines and reducing protective adipokines like adiponectin.^82^ In obesity, decreased sensitivity to leptin (*LEP*), a key adiponectin involved in energy balance and hunger inhibition, often leads to leptin resistance, decreased satiety and increased body mass.^83^ Both human and mouse platelets express the leptin receptor (*LEPR*, *Lepr*), and leptin promotes ADP‐induced platelet aggregation, and leptin‐deficient mice show a reduced thrombotic response, linking obesity to thrombosis.^84^, ^85^

Dysfunctional adipose tissue also induces systemic oxidative stress and ROS accumulation. Regarding platelets, increased ROS production enhances lipid peroxidation, measurable through specific urinary TXA_2_ metabolites like 8‐iso‐PGF_2α_ and 11‐dehydro‐TxB. These metabolites not only serve as oxidative stress markers but also directly amplify platelet activation, potentially interacting with the thromboxane receptor, as reviewed in Ting and Khasawneh.^19^

### Platelet communication with the vessel wall in obesity

Platelet miRNAs, mRNAs and vesicles are increasingly recognized as significant factors in obesity. In the Framingham study, inflammatory platelet‐derived transcripts were linked to obesity and coronary heart disease.^86^ Young obese females show altered platelet RNA content as compared with matched normal‐weight controls, including elevated levels of cardiovascular risk markers protein S100A9 (*S100A9*) and AGER (*AGER/RAGE*).^87^ Upon bariatric surgery, platelet RNA expression was altered,^87^, ^88^ with an unclear effect on platelet function ranging from a reduction in inducible platelet P‐selectin expression^87^ to unaltered platelet aggregation.^88^

The idea that platelet‐derived transcripts can impact end‐organ functions was elegantly shown in a recent study by Cariello *et al*. Here, platelet‐derived miR‐19a was suggested to mediate colon cancer development in a setting of visceral obesity.^89^ Moreover, oxLDLs have been reported as an important promotor of PMV release, and increased vesicle overproduction in obesity may be partially reverted through the reduction of body mass.^90^

### Obese individuals poorly respond to antiplatelet therapy

Obese individuals display an impaired response to antiplatelet medication, partly because of altered pharmacokinetics, reflecting the variation in body mass and drug metabolism. Platelet hyperactivity resulting from enhanced thrombopoiesis, oxidative stress and inflammation may contribute to this reduced effectiveness of antiplatelet drugs.^91^ Recent findings showing obese animals having up‐regulated procoagulant platelets^81^ raise the question whether targeting this specific prominent platelet population in obesity could be a promising avenue for future antiplatelet therapy.

## Platelets in DM

### DM

DM is a chronic disease responsible for the death of 1.5 million people in 2019,^68^ with around 30% prevalence in HFpEF.^5^ Over 70% of DM‐related deaths are linked to CVD, with DM patients having a two‐fold to four‐fold increased risk of heart attacks and strokes.^92^ Diabetes is associated with accelerated atherosclerosis, which underlies macrovascular complications like CAD, peripheral vascular disease and stroke. Moreover, DM is associated with microvascular complications such as retinopathy, neuropathy and nephropathy.^92^ Chronic vessel damage can result in organ dysfunction and failure, affecting the kidneys, eyes and heart.^92^ Renal dysfunction is associated with HFpEF but this is beyond the scope of this review.^5^

### The ‘diabetic platelet’: Altered production and phenotype

Over the years, numerous studies have revealed altered platelet activity in DM patients, leading to the characterization of a specific phenotype termed ‘diabetic platelet’. These patients show changes in platelet production, including increased megakaryocyte ploidy^93^ and a higher number of reticulated platelets, indicating accelerated platelet turnover.^94^ The proposed mechanism involves elevated blood glucose activating neutrophils, leading to the release of S100A8/A9 proteins, which stimulate thrombopoietin (TPO, *THPO*) production upon interacting with the RAGE receptor on Kupffer cells, resulting in increased thrombocytosis.^95^ The impact of this altered platelet synthesis on platelet morphology and function is an open area of investigation.

Diabetic platelets are larger,^96^ hyperactive and functionally altered. Platelets from diabetic individuals display increased platelet glycoprotein GPIb and αIIbβ3 expression, an overexpressed and constitutively activated P2Y_12_ receptor, as well as increased degranulation, as reviewed by Santilli *et al*.^97^ Recent findings also indicate the up‐regulation of platelet ion channel Piezo1 activity in chronic hyperglycaemia, triggering prothrombotic responses including platelet intracellular calcium elevation, increased PS exposure and platelet‐induced thrombin generation.^98^

### High glucose levels contribute to platelet hyperactivation

**Figure 1:**
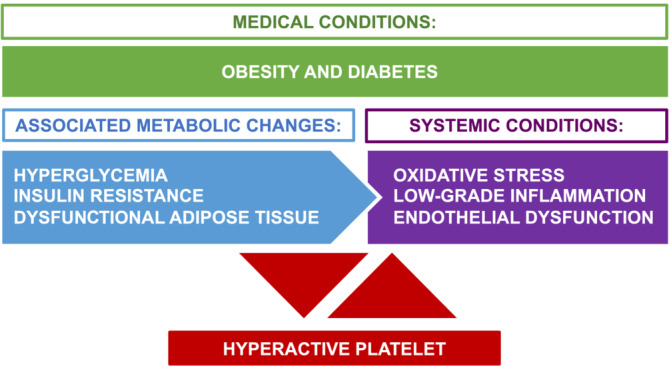
Scheme of platelet hyperactivity in diabetes mellitus and obesity. Hyperglycaemia, insulin resistance, dysfunctional adipose and associated increased oxidative stress, systemic inflammation and endothelial dysfunction contribute as causal factors in platelet hyperactivity in diabetes and obesity.

DM is associated with metabolic changes such as hyperglycaemia and insulin resistance that contribute as casual factors in platelet hyperactivity observed in these patients (see Figure 1). Hyperglycaemia, that is, high blood glucose, is the result of inadequate insulin production or activity and can facilitate platelet hyperactivation in multiple ways with a central role for increased platelet ROS production herein, as reviewed in detail by Santilli *et al*.^97^ Platelets express the insulin receptor,^99^ and human platelets are subject to insulin resistance in DM.^100^ Insulin has been suggested to mitigate platelet responses to multiple agonists through the enhancement of NO and cGMP levels with consequent reduction in intracellular calcium levels, thus inhibiting activation. Importantly, cell‐reduced sensitivity to insulin linked to DM can disrupt these pathways, enhancing platelet responsiveness and potentially increasing thrombotic risk.^100^ However, a more recent study observed that deleting the insulin receptor in mouse megakaryocytes and platelets did not induce platelet hyperactivity, albeit platelet count and volume were increased, suggesting that the enhanced platelet activity linked to insulin resistance is unlikely due to the compromised insulin receptor signalling but to the modifications in platelet count and volume parameters promoted by megakaryocyte/platelet insulin resistance in obesity and type 2 DM (T2DM).^101^

### Low‐grade systemic inflammation, oxidative stress and platelet function

Low‐grade systemic inflammation is thought to promote platelet activation in DM.^102^ Vice versa, platelets may also contribute to this inflammatory environment because they contain numerous inflammatory factors that, once platelets are activated, can be released in circulation. Examples are enhanced levels of circulating platelet‐derived LIGHT (*TNFSF14*), CD36 (platelet glycoprotein 4, *CD36*) and DKK‐1 (*DKK1*) in diabetics, as reviewed in Santilli *et al*.^97^

As mentioned above, DM is also associated with enhanced oxidative stress due to higher ROS levels and nitrogen species production.^103^ ROS promote platelet protein nitration and inactivation, for example, of the sarcoendoplasmic reticulum Ca^2+^ ATPase 2 (SERCA‐2, *ATP2A2*), with implications for platelet calcium levels.^104^ The interplay of inflammation and oxidative stress promotes CD40L up‐regulation, which consequently enhances platelet activation.^105^ Furthermore, diabetes‐associated glycation of circulating albumin, when incubated in vitro with platelets, increases platelet aggregation and hyperactivity.^106^

### Impaired platelet–vessel wall interactions in DM

Diabetic platelets and megakaryocytes show altered miRNA expression profiles, with reduced levels of specific miRNAs such as miR‐223,^107^, ^108^ involved in P2Y_12_ receptor expression, miR‐146a,^108^ miR‐26b, miR‐126 and miR‐140.^107^ Interestingly, miR‐103, important for glucose homeostasis and insulin sensitivity,^109^ is enriched in human platelets.^110^ The down‐regulation of platelet‐derived miR‐103b was identified as a potential biomarker for the pre‐diabetic state.^111^

The theory behind altered miRNA expression in DM involves up‐regulated calpain, leading to proteolytic degradation of the endonuclease Dicer, impairing precursor miRNA maturation.^112^ Furthermore, T2DM platelets contain fewer pre‐miRNAs, possibly due to reduced levels in hyperglycaemia‐exposed precursor megakaryocytes.^107^

PMVs have been shown to promote endothelial dysfunction in DM, as calpains delivered from platelets to endothelial cells via PMVs cleave the N‐terminus of the PAR‐1 receptor on endothelial cells, promoting vascular inflammation and increasing ICAM‐1 expression and TNF‐α release.^113^

### DM and suboptimal response to antiplatelet medications

Secondary prevention strategy in patients with diabetes and a history of CVD recommends the administration of low‐dose aspirin. However, these patients often show a suboptimal response to antiplatelet medication, including aspirin and clopidogrel. DM‐associated oxidative stress potentially impairs the anti‐aggregating effect of aspirin as well as aspirin‐dependent cyclooxygenase (COX) acetylation, compromising the anti‐inflammatory effects of aspirin.^114^ The accelerated platelet turnover that characterized these patients may also contribute through the presence of younger and more reactive platelets that are less sensitive to medications.^97^ Despite these theories, mechanisms are still largely unknown.

## Platelets—Age and sex

### Platelet characteristics and function during ageing

HFpEF is prevalent in the elderly, who are at increased risk of CVDs such as HF, atherosclerosis and vascular stiffening.^115^ Also, platelets are affected in multiple ways as we age. Platelets from adults are enriched in proteins related to immune functions when compared with neonatal platelets, which contain more proteins involved in metabolic activities.^116^ Platelet count decreases with age in both sexes, with aged females experiencing a 25% reduction and males a 35% reduction compared with early childhood levels.^117^ This reduction may be linked to decreased haematopoietic stem cells during ageing.^118^ MPV increases during ageing,^119^, ^120^ and platelet activity may also increase, as evidenced by enhanced agonist‐induced platelet aggregation.^121^, ^122^ Furthermore, ageing is associated with increased oxidative stress, which is involved in platelet activation. Old mice showed increased platelet adhesion during thrombus formation and elevated levels of intraplatelet hydrogen peroxide, which is responsible for enhanced platelet integrin αIIbβ3 activation and fibrinogen binding.^123^ Moreover, a study showed that aged mice display increased megakaryocyte size, MPV and higher venous thrombosis risk compared with young mice.^124^ Ageing‐associated inflammation, or inflammageing, may further contribute to platelet activation and mitochondrial dysfunction. In particular, TNF‐α‐driven metabolic reprogramming of megakaryocytes and high TNF‐α levels in platelets from aged mice are associated with critical changes in mitochondria morphology and activation, contributing to altered platelet activation and increased thrombosis risk.^125^ Moreover, superoxide dismutase 2 (*Sod2*) deficiency in mice amplifies age‐related platelet hyperactivation and thrombotic risk.^126^ Interestingly, in both mice and humans, platelet redox regulation appears non‐linear during ageing, with individuals over 80 years showing an adaptive increase in platelet antioxidants, resulting in decreased platelet activity,^127^ which could have implications for antiplatelet therapy in individuals >80 years old. In the past, platelet markers β‐thromboglobulin and platelet factor 4 (C–X–C motif chemokine 4, *PF4/CXCL4*) were found to increase in old individuals.^128^ However, a recent study shows decreased PF4 levels in plasma from both old mice and humans. Moreover, systemic administration of PF4 in aged mice reduced hippocampus inflammation and promoted synaptic plasticity, highlighting the potential of PF4 as a pro‐cognitive factor for treating age‐related neurodegenerative diseases.^129^

### Platelets in females and the role of menopause

HFpEF is characterized by female predominance. Research, as reviewed by Sabetta *et al*.,^130^ suggests higher platelet reactivity in women compared with men, although this remains debated. Healthy females also have higher platelet counts than males.^131^ However, increased platelet responsiveness in women is thought to be an intrinsic feature rather than solely a result of a higher count.^130^, ^132^ Upon ageing, alterations in von Willebrand factor (vWF)‐related platelet functions appear more profound in women compared with men, including decreased platelet translocation and unstable interactions on vWF.^133^

As compared with men, platelets from healthy females show a higher number and more activatable integrin αIIbβ3, crucial in thrombus formation.^134^ In the Framingham Heart Study, women had significantly higher platelet levels of toll‐like receptor mRNAs associated with prothrombotic P‐selectin.^135^ Moreover, platelets isolated from healthy young women show an increased aggregation in response to PAR‐1 and thromboxane receptor agonists.^136^ Upon correcting for platelet count, unaltered ADP‐induced platelet reactivity was observed in post‐menopausal females versus males also undergoing cardiac surgery.^132^ Recently, a study observed species‐conserved sex differences in platelet activation before and following an MI with platelet protease‐activated receptor (PAR) signalling decreasing in women and increasing in men after MI, opposite to healthy conditions.^137^ This study highlights sex‐specific platelet signalling as well as changes between healthy and pathological conditions. Results from the large population‐based Gutenberg Health Study of over 15 000 subjects show sex differences in genetic and non‐genetic determinants of platelet size (MPV) and its association with total mortality. In females, the use of oral contraceptives and menstruation are strongly associated with higher MPV, while only in males is higher MPV linked to age and cardiovascular risks, including hypertension and high glucose levels, and a higher risk of death.^138^ Sex hormones also play a crucial role in menopause, where declining oestrogen and progesterone levels reduce their cardioprotective effects. Platelets express both the oestrogen receptor β (*ESR2*) and the androgen receptor (*AR*). Studies in aged female mice lacking the oestrogen β receptor show increased reticulated platelets and procoagulant microvesicles, enhanced thrombin generation and platelet mitochondrial dysfunction.^139^, ^140^

The decline in oestrogen levels during menopause may contribute to the pathophysiology of HFpEF.^141^ Ongoing research explores the potential benefits of menopausal hormone therapy and synthetic 17β‐oestradiol (E2) in preventing cardiovascular risks associated with menopause.^142^ However, studies on the impact of oestrogen administration on platelet activation display contradictory results, with some showing increased platelet receptor expression and PMV release after therapy, while others observing no difference or reduced platelet activation, as reviewed by Dupuis *et al*.^143^

#### Antithrombotic medication in men and women

Historically, sex differences were often overlooked in studies of CVD pathophysiology and treatment, with women underrepresented in clinical trials. However, there is growing recognition of this issue, with studies now focusing on sexual differences and stratification. This represents a crucial step towards personalized and more effective medical interventions.

Females may experience reduced benefit from antiplatelet medications, showing higher platelet reactivity after dual‐antiplatelet or low‐dose aspirin therapies compared with males, although the difference may not be sufficient to identify patients with a higher risk of a worse prognosis.^144^, ^145^ Additionally, research suggests that in women treated with clopidogrel, higher platelet reactivity induced by ADP may be due to differences in platelet count rather than function.^132^ Evidence also indicates a potential association between aspirin usage and increased occurrence of heavy menstrual bleeding in women.^146^

## Altered platelet phenotype in HFpEF and its comorbidities: A larger perspective

This review aims to provide an in‐depth overview of platelet role in HFpEF and its major comorbidities. Normally, the endothelium prevents platelet activation by releasing antiplatelet agents like NO and PGI_2_, maintaining blood flow and preventing clot formation. However, HFpEF and its comorbidities often exhibit impaired platelet response to these agents, of which causes are often unknown. The development of platelet resistance to these inhibitors may lead to uncontrolled platelet activation and increased prothrombotic risk. Understanding the molecular mechanisms underlying this resistance could offer insights into preventing or treating CVDs.

**Figure 2:**
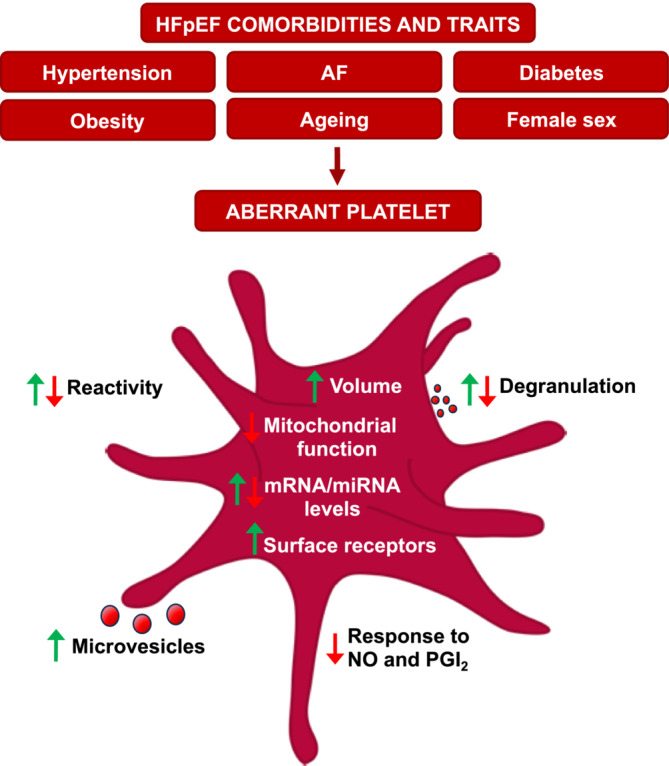
Platelet alterations in heart failure with preserved ejection fraction (HFpEF) comorbidities and traits. HFpEF comorbidities present alterations in platelet behaviour and in features linked to platelet content and reactivity, including reduced response to nitric oxide (NO) and prostaglandin I_2_ (PGI_2_), increased surface receptor expression and microvesicle release, mitochondrial dysfunction and transcriptome alterations. AF, atrial fibrillation; miRNA, microRNA.

All the HFpEF comorbidities are associated with alterations in platelet features linked to platelet content and reactivity (Figure 2). Parameters linked to platelet size are increased in most comorbidities, and MPV was recently linked to worse clinical outcomes in HFpEF itself.^20^, ^21^ Total platelet count is also often altered in HFpEF comorbidities; female sex and obesity are associated with increased platelet count, whereas AF and ageing show the opposite trend. Reticulated platelets, defined as young, large and RNA‐rich, are also increased in most HFpEF‐associated conditions. Recently, a strong interest in the concept of platelet heterogeneity is emerging, which means the presence of different platelet populations, consisting of platelets with different characteristics linked to their size, lifespan and expression of surface markers. These differences may also lead to functional changes. New methods like multicolour flow cytometry are aiding in understanding these platelet subtypes, offering insights into their roles in pathological contexts.

**Figure 3:**
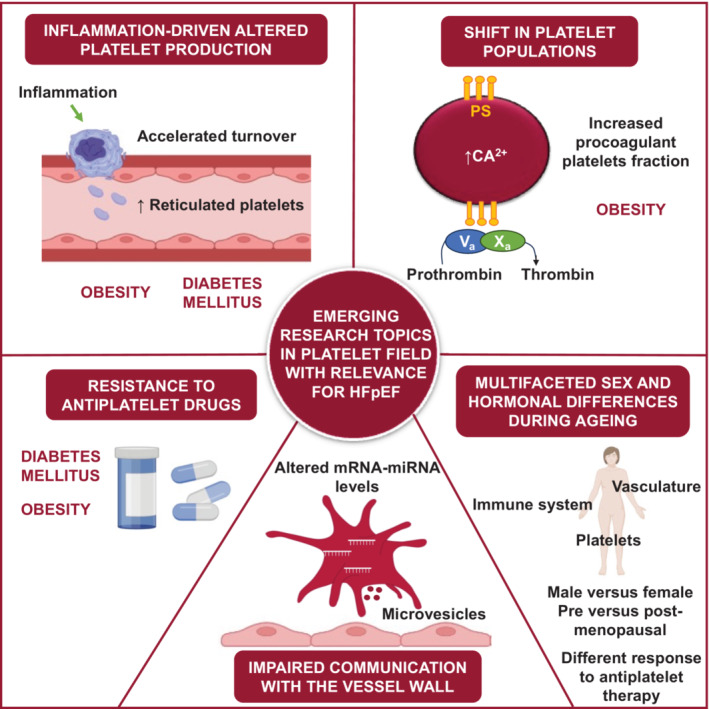
Emerging research topics in platelet biology in the context of heart failure with preserved ejection fraction (HFpEF) and HFpEF comorbidities. These include exploring changes in platelet production, discriminating distinct platelet populations in HFpEF, investigating the mechanisms underlying platelet interactions with the vessel wall and patient resistance to antiplatelet therapy, and exploring sex differences. miRNA, microRNA; PS, phosphatidylserine.

Despite extensive studies on platelet biology in CVDs, there are still unexplored areas, as illustrated in Figure 3. One of the themes is platelet production. Ageing is associated with decreasing platelet count, which is possibly caused by a reduction in haematopoietic stem cells.^118^ CVDs like diabetes also show dysfunctional platelet production and accelerated platelet turnover.^95^ Adipose tissue was also found as a player in platelet production in obese mice.^73^ A recent study highlights the role of neutrophils in triggering accelerated platelet production, increasing the portion of reticulated platelets, upon MI.^147^ Understanding platelet production in HFpEF is challenging due to a lack of circulating markers and reliance on patient blood samples in the absence of good preclinical models.^148^ Furthermore, whether platelet production differs between sexes and its impact on other comorbidities remains to be established. Additionally, the implications of reduced platelet energy production and mitochondrial function observed in diabetes, HFpEF and ageing require further investigation.

In HFpEF comorbidities, there is often an increase in surface receptor expression, soluble factor release from granules and circulating PMVs. These changes may influence platelet–vessel wall interactions, contributing to endothelial inflammation and dysfunction. PMV‐mediated transfer of various factors including miRNAs can induce morphological and functional changes in the endothelium in vitro.^149^ Further understanding of this platelet RNA‐mediated mechanism may unveil new therapeutic targets. While alterations in platelet mRNA and miRNA expression profiles are observed in many comorbidities, possibly leading to altered protein expression both in platelets and in recipient cells, their specific impact on HFpEF and vascular function remains unexplored. Advancements in technology will help in elucidating these mechanisms and their relevance to disease progression.

## Conclusions

HFpEF presents a complex and still partially unexplored pathophysiology. We propose that platelets play a significant role in this context. Altered platelet phenotype and function are observed in most HFpEF‐associated comorbidities, with early studies suggesting dysfunctionality in HFpEF itself, though this remains to be confirmed. Inflammation and oxidative stress can contribute to platelet hyperactivation, and once activated, platelets can release numerous factors that can further enhance these processes. With that, platelets may be involved in the initiation and development of the disease. Various aspects linked to platelet characteristics, reactivity and content can be compromised, affecting vascular integrity. However, numerous aspects remain unexplored, and the scarcity of scientific literature on platelet function specifically related to HFpEF is a challenge to overcome.

## Conflict of interest statement

The authors declare that they have no conflicts of interest.

## Funding

Judith M.E.M. Cosemans is supported by the Netherlands Organization for Scientific Research [Nederlandse Organisatie voor Wetenschappelijk Onderzoek (NWO); Vidi 91716421] and by the European Union's Horizon 2020 Research and Innovation Program under the Marie Skłodowska‐Curie Grant Agreement No. 813409. Judith M.E.M. Cosemans and Blanche Schroen are funded by a BHF‐DZHK‐DHF International Cardiovascular Research Partnership Award (02‐001‐2022‐0124).
